# Does the family affluence scale reflect actual parental earned income, level of education and occupational status? A validation study using register data in Sweden

**DOI:** 10.1186/s12889-021-11968-2

**Published:** 2021-11-03

**Authors:** Maria Corell, Yun Chen, Peter Friberg, Max Petzold, Petra Löfstedt

**Affiliations:** 1 School of Public Health and Community Medicine, Institute of Medicine, Gothenburg University, Box 463, 405 30 Göteborg, Sweden; 2 Public Health Agency of Sweden, 171 82 Solna, Sweden

**Keywords:** Family affluence scale, Validation, Sweden, Register data, HBSC, STARS

## Abstract

**Aim:**

To examine the external validity of the Family Affluence Scale (FAS) among adolescents in Sweden by using register data for parental earned income, level of education and occupational status.

**Methods:**

Data from the baseline (2015–2019) of the Study of Adolescence Resilience and Stress (STARS), comprising 2283 13-year-olds in the region of Västra Götaland, were used. The FAS III consists of six items: unshared bedroom, car ownership, computer/tablet ownership, dishwasher, number of bathrooms and number of holidays abroad. Register data regarding earned income, educational level and occupational status from Statistics Sweden (2014–2018) were linked to adolescents. In total, survey data were available for 2280 adolescents, and register data were available for 2258 mothers and 2204 fathers.

**Results:**

Total parental earned income was moderately correlated with adolescents’ scoring on FAS (0.31 < *r* < 0.48, *p* < 0.001), depending on examination year. The low FAS group mainly comprised low-income households, and the high FAS group mainly comprised high-income households. Correlations between mothers’ and fathers’ educational level and adolescents’ scoring on FAS were low (*r* = 0.19 and *r =* 0.21, respectively, *p* < 0.001). FAS was higher among adolescents whose parents were working, but the correlation between parents’ occupational status and FAS was low (*r =* 0.22, *p* < 0.001).

**Conclusions:**

The FAS can mainly identify low- and high-income households in Sweden. It may be used as an alternative measure of parental earned income in studies using self-reported socioeconomic status among adolescents.

## Introduction/background

Socioeconomic conditions early in life shape future inequalities in social development, education, employment and adult health. Consequently, the social gradient in health is found across the life cycle, in early childhood, during adolescence and among adults. It runs from top to bottom of the socioeconomic spectrum, which means that health inequities affect everyone [1]. In rich countries, where absolute deprivation is low, the social gradient shifts to relative deprivation [2]. Reliable indicators of both absolute and relative socioeconomic conditions are therefore crucial in all epidemiological research.

There are difficulties when measuring socioeconomic conditions using self-report surveys among children and adolescents. Commonly used indicators of socioeconomic status in epidemiological studies are education, income or occupation. However, children may not be able to provide their parents’ occupation or education accurately, especially younger children [3] and those from families with low socioeconomic status [4, 5]. This leads to low completion rates and a risk of systematically missing values, creating bias in the results. For these reasons, the Family Affluence Scale was developed.

### Development of the family affluence scale

The FAS was developed in Scotland at the beginning of the 1990s and included three items on material conditions in the family: telephone ownership, car ownership and unshared bedroom [3]. The scale was based on the work of Townsend [6] and Carstairs and Morris [7]. The FAS was adopted by the international Health Behaviour in School-aged Children (HBSC) study in the 1993/94 data collection; however, the telephone item was omitted, and only the items regarding car ownership and unshared bedroom were included [5].

Since then, the HBSC network has continuously revised the scale due to improved living conditions, technical innovations, and changing patterns of consumption and lifestyle. In 1997/98, a question regarding the number of family holidays was included (FAS I). In 2001/02, an item on the number of computers was added (FAS II). In 2013/14, two more items were added as a result of a cross-national study of potentially new items in eight countries: dishwasher and number of bathrooms. Additionally, the holiday item was changed to holidays *abroad* (FAS III) [8].

### Using the FAS in research

The FAS is frequently used in international research examining socioeconomic inequalities in health among children and adolescents. In the international WHO/HBSC report, published after each HBSC data collection, all indicators are presented by age, gender and family affluence (see [9]). Comparisons of low- and high-affluence groups with regard to health behaviours and health outcomes, based on international HBSC data, show increasing health inequalities over time [10]. The FAS can also be used to measure relative affluence in schools and regions [11]. As the HBSC is a unique study of children’s and adolescents’ living conditions, health behaviours and health that has been ongoing since 1985/86 in Sweden and is now ongoing in 50 countries, there is constantly a need to ensure that the items measuring families’ socioeconomic conditions are still reliable and valid.

### Previous validation work

The FAS has been validated in several countries in Europe, Asia and North America. In several validation studies, internal reliability was examined using Cronbach’s alpha. In seven studies, the external validity was examined by comparisons of children’s responses to FAS and their reports of parents’ occupation or education [3, 4, 12–16]. In three validation studies, the conformity of children’s responses to FAS with their parents’ responses to FAS or reports about their income was examined [8, 17, 18]. In four studies, the functioning of FAS between different countries and over time was investigated [8, 19–21]. In three studies, the FAS was validated using macro level indicators, such as GDP [22], regional disposable income [23] and the area deprivation index [24].

In summary, six studies showed low to moderate external validity [3, 4, 12, 14–16], while four showed high external validity [8, 13, 17, 18].

### Knowledge gaps

We found one validation of the FAS II conducted in Sweden [16], where the external validity of the scale was examined using children’s reports of parental occupation. However, the internal reliability and the scale’s conformity with other measures of SES, such as parental education or income, have not been examined in Sweden. Furthermore, we found only two validation studies conducted on FAS III, i.e. the version of the FAS that has been included in the HBSC study since 2013/14 [8, 23]. While the study by Torsheim et al. [8] used parents’ scoring on FAS and parental reported income as external validation criteria, the study by Hobza et al. [23] validated aggregated FAS per region against the regional disposable income per capita. Consequently, there is a need to examine whether the FAS is still valid since the FAS is an asset-based indicator and societal patterns of consumption and family lifestyles constantly change. We have not found any validation study from Sweden or any other country that has used register data to validate FAS despite register data being more reliable than both child-reported and parent-reported data on income, education and occupation.

In the STudy of Adolescence Resilience and Stress (STARS), the FAS is included in the questionnaire to adolescents. Register data from Statistics Sweden regarding, e.g., the parents’ level of education, earned income and occupational status, were linked to the adolescents in the study. This means that survey data from STARS together with register data can be used to validate FAS among 13-year-olds in Sweden. This can be done by examining whether the children’s responses to FAS are associated with their parents’ earned income, level of education and occupational status.

## Aims

The overall aim of this study is to examine the external validity of the Family Affluence Scale (FAS) among adolescents in Sweden by using register data for parental earned income, level of education and occupational status.

The first research question is whether there is an association between parents’ socioeconomic status (earned income, level of education and occupational status) and adolescents’ responses to FAS. The second research question is whether *current HBSC guidelines* for categorizing adolescents into low, medium and high FAS are applicable in Sweden.

## Methods

### Data

Data from the baseline (2015–2019) of the STARS study, comprising 2283 13-year-olds from 54 schools in 16 municipalities in the region of Västra Götaland, were used. Schools were selected from areas with various socioeconomic contexts. With consent from the principals, researchers visited 7th grade classes to inform students and their teachers about the study. Next, information letters were sent to students and their parents or guardians. Informed consent from both students and parents (or guardians) was obtained before participation. The response rate was 45%. Ethical approval was obtained by the Regional Ethics Board in Gothenburg in August 2015 (Dnr 578–15).

The students completed a questionnaire and participated in physical examinations of e.g. height, weight, blood pressure, heart rate and submitted hair samples for cortisol analyses. Register data from Statistics Sweden were added to the STARS study in 2020. Ethical approval was obtained from the Swedish Ethical Review Authority in December 2019 (Dnr 2019–06035) for this purpose. In total, survey data were available for 2280 students (55.6% girls, aged 13.6 ± 0.4 years), and register data were available for 2258 mothers and 2204 fathers.

### Variables

*The Family Affluence Scale III* consists of six items regarding the family’s material assets: number of cars (0, 1, 2 or more), number of bathrooms (0, 1, 2, 3 or more), number of computers (0, 1, 2, > 2), unshared bedroom (no/yes), dishwasher (no/yes) and the number of holidays abroad during the last 12 months (0, 1, 2, 3 or more). The children’s answers to the six items were summed up (0–13). The international guidelines [25, 26] state that for each country participating in the HBSC, age-group and gender-specific ridit scores should be calculated. The ridit transformation was developed by Bross [27] and is the basis for computing regression-based indicators of socioeconomic inequality. The ridit scores are then used to identify groups of children and adolescents in the lowest 20% (low affluence), middle 60% (medium affluence) and highest 20% (high affluence). For this sample, this means that the following cut-offs should be used for both boys and girls: 0–7 (low), 8–11 (medium) and 12–13 (high). In this study, the FAS was used both as a continuous variable (0–13) and divided into low, medium and high family affluence.

*Earned income* (for mother and father, respectively, and together) includes both earned income from employment and income from self-employment. It also includes pensions, sickness benefits, parental allowances, unemployment benefits, etc. It does not include child benefits, housing allowances or income support. The information comes from the Income and Taxation Register at Statistics Sweden. In this study, we used the mother’s and father’s income during the year *preceding* the year the student completed the STARS questionnaire. Because income generally increases every year, the variable was divided into quintiles for each year (2014–2018) to make data from different years comparable. The variable was also used as a continuous variable in the analyses. Data were available for 2213 mothers, 2141 fathers and 2116 parents (see Table 1).

**Table 1:** Parental level of earned income, level of education and occupational status (2014–2018) in STARS

Variable	*n*	%
*Mother’s Earned Income*		
Quintile 1	*445*	20.1
Quintile 2	*449*	20.3
Quintile 3	*440*	19.9
Quintile 4	*442*	20.0
Quintile 5	*437*	19.7
Total	*2213*	100.0
*Father’s Earned Income*		
Quintile 1	*429*	20.0
Quintile 2	*430*	20.1
Quintile 3	*428*	20.0
Quintile 4	*421*	19.7
Quintile 5	*433*	20.2
Total	*2141*	100.0
*Total Parental Earned Income*		
Quintile 1	*425*	20.1
Quintile 2	*424*	20.0
Quintile 3	*422*	19.9
Quintile 4	*424*	20.0
Quintile 5	*421*	19.9
Total	*2116*	100.0
*Mother’s Highest Level of Education*		
Primary School	*151*	6.9
Secondary School	*718*	32.6
Post-secondary school	*1334*	60.6
Total	*2203*	100.0
*Father’s Highest Level of Education*		
Primary School	*168*	8.7
Secondary School	*805*	41.7
Post-secondary school	*957*	49.6
Total	*1930*	100.0
*Parents’ Occupational Status*		
No parent works	*57*	2.7
One parent works	*259*	12.2
Both parents work	*1800*	85.1
Total	*2116*	100.0

*The highest level of education* (for mother and father, respectively) shows the parents’ highest level of education. There were originally seven levels available in the Education register at Statistics Sweden, and in this study, they were aggregated to the following three groups: 1) 9 years or less, 2) 11–12 years, and 3) post-secondary education. This means that education levels are primary school, secondary school and post-secondary school. These three levels of education are often used in research. However, the original seven levels of education available in registers were also analysed to examine whether correlation coefficients differed from the three levels of education. The parents’ level of education in the year *preceding* the year the student completed the STARS questionnaire was used in this study. Data were available for 2203 mothers and 1930 fathers.

*Occupational status* shows whether the parents were working or not. In this study, three categories were used: 1) none of the parents were working, 2) one parent was working and one not, and 3) both parents were working. Both employees and self-employed individuals were included in the second two categories. In this study, the parents’ occupational status in the year *preceding* the year the student completed the STARS questionnaire was used. Data were available for 2116 parents.

### Statistical methods

Cronbach’s alpha was calculated for FAS. A Principal Component Analysis was performed to examine if the scale was unidimensional. As the FAS sum scores were not normally distributed, median values and interquartile ranges (IQRs) were calculated for each earned income quintile, level of education and parental occupational status. Kruskal-Wallis tests were performed to determine whether differences in medians across groups were statistically significant (*p* < 0.05).

Pearson’s correlation coefficients were calculated for parental earned income (in SEK) and FAS sum scores. Spearman’s correlation coefficients were calculated for parental level of education and FAS sum scores and for parental occupational status and FAS sum scores. Correlation coefficients (*r*) were interpreted as follows: 0–0.29 as low, 0.30–0.49 as moderate and 0.5–1.0 as high correlation [28]. All analyses were performed in IBM SPSS version 26.0.

## Results

**Fig. 1:**
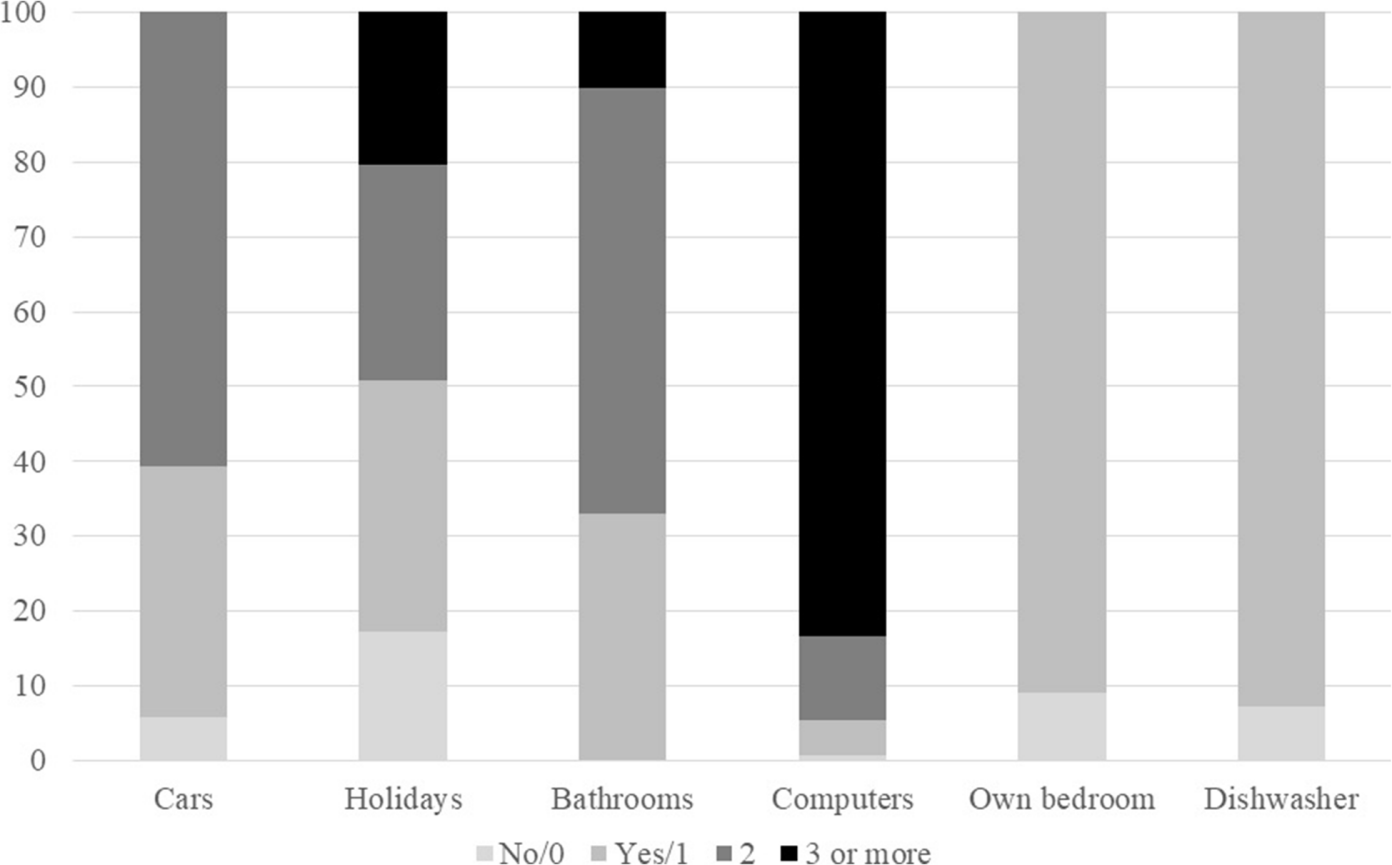
Adolescents’ responses to each item on the FAS, STARS 2015–2019

Figure 1 shows the adolescents’ responses to each item on the FAS. As seen from the figure, all adolescents stated that they had at least one bathroom at home. Almost all children (99.3%) stated that the family had a computer (PC, Mac, laptops, tablets and Ipads), of which most stated that the family had three or more. 94.1% stated that the family had at least one car, 92.7% that they had a dishwasher at home and almost as many (90.9%) that they had an unshared bedroom. The majority, 82.7%, had been on holiday abroad during the past 12 months.

Cronbach’s alpha for the scale was 0.516. Omitting the holiday item would increase Cronbach’s alpha marginally to 0.535. Deleting any of the other items would decrease the Cronbach’s alpha, especially deleting the car ownership item (α = 0.408). Inter-item correlations were low, ranging from 0.060 to 0.307.

The Principal Component Analysis showed that the scale was unidimensional and that 33.36% of the total variance was explained by the component. The KMO and Bartlett’s Test showed a value of 0.729 (*p* < 0.000).

Before the research questions were addressed, several steps were taken to determine if the students who participated in STARS were representative of Sweden, even though they constituted a regional sample.

First, comparisons with 13-year-olds in the nationally representative HBSC 2017/18 were carried out, showing that the proportion with a foreign background was slightly smaller in STARS, while the proportion who lived with both parents was slightly larger.

Second, the children’s scoring on the FAS in STARS was compared with 13-year-olds’ scoring on the FAS in HBSC 2017/18. As Table 2 shows, the distribution of FAS was similar in STARS and HBSC 2017/18. Furthermore, the mean value of FAS was 9.45 ± 1.97 in STARS compared to 9.41 ± 2.00 in HBSC. Cronbach’s alpha was 0.516 in STARS compared to 0.453 in HBSC.

**Table 2:** Adolescent’s scoring on the Family Affluence Scale in STARS 2015–2019 and HBSC 2017/18

FAS	STARS		HBSC	
score	%	***n***	%	***n***
1	0	*0*	0.2	*3*
2	0.2	*5*	0.4	*6*
3	0.7	*15*	0.2	*3*
4	0.9	*21*	0.5	*7*
5	2.0	*45*	2.7	*37*
6	4.8	*109*	3.6	*50*
7	6.5	*149*	7.2	*100*
8	12.1	*275*	13.3	*185*
9	19.0	*434*	21.0	*291*
10	21.7	*495*	20.9	*289*
11	18.1	*413*	16.2	*224*
12	11.5	*262*	9.0	*125*
13	2.5	*57*	4.8	*66*
Total	100.0	*2280*	100.0	*1386*

Finally, the level of education among parents in STARS was compared to the education level among 35–44-year-olds in the Swedish population according to the Education register at Statistics Sweden. Comparisons showed that parents in STARS, especially fathers, were slightly more educated than the population in general.

Overall, the differences between children and parents in STARS, HBSC 2017/18 and register data at Statistics Sweden were small. This indicates that the respondents in STARS were representative of the country as a whole.

### Associations between parents’ earned income and FAS

First, the association between parental earned income and adolescents’ scoring on the FAS was examined. As the results in Table 3 show, it is clear that the median value of FAS gradually increased with increased parental earned income. The median value of FAS was 9 among adolescents whose mothers belonged to the lowest earned income quintile and then gradually increased to 11 among adolescents whose mothers belonged to the highest earned income quintile. The Kruskal-Wallis test showed significant differences in FAS across income quintiles (235.39, *p* < 0.001).

**Table 3:** Parental level of earned income, level of education and occupational status (2014–2018) and adolescents’ responses to the Family Affluence Scale (2015–2019)

	Median FAS	Inter Quartile Range (IQR)	*n*	Kruskal-Wallis Test
*Mother’s Earned Income*				
Quintile 1	9	6–12	445	
Quintile 2	9	7–11	449	
Quintile 3	10	8–12	440	235.39
Quintile 4	10	8–12	442	*p* < 0.001
Quintile 5	11	9–13	437	
*Father’s Earned Income*				
Quintile 1	9	6–12	429	
Quintile 2	9	7–11	430	
Quintile 3	10	8–12	428	295.98
Quintile 4	10	8–12	421	*p* < 0.001
Quintile 5	11	9–13	433	
*Total Parental Earned Income*				
Quintile 1	9	6–12	425	
Quintile 2	9	6–12	424	
Quintile 3	10	8–12	422	327.13
Quintile 4	10	8–12	424	*p* < 0.001
Quintile 5	11	9–13	421	
*Mother’s Highest Level of Education*				
Primary School	8	5–11	151	
Secondary School	9	6–12	718	96.18
Post-secondary school	10	8–12	1334	*p* < 0.001
*Father’s Highest Level of Education*				
Primary School	9	6–12	168	
Secondary School	9	6–12	805	85.15
Post-secondary school	10	8–12	957	*p* < 0.001
*Parents’ Occupational Status*				
No parent works	7	4–10	57	
One parent works	9	6–12	259	110.14
Both parents work	10	8–12	1800	*p* < 0.001

The results were similar for fathers’ level of earned income; the median value of FAS was 9 among adolescents whose fathers belonged to the lowest earned income quintile and gradually increased to 11 among adolescents whose fathers belonged to the highest earned income quintile. The Kruskal-Wallis test showed significant differences in FAS across income quintiles (295.98, *p* < 0.001).

The same pattern was found for total parental earned income, where median values of FAS gradually increased from 9 to 11. The Kruskal-Wallis test showed significant differences in FAS across income quintiles (327.13, *p* < 0.001).

The results in Table 3 were confirmed when Pearson’s correlations were performed, with parental earned income as a continuous variable together with the FAS as a continuous variable (0–13); see Table 4. The correlation coefficients (*r*) for fathers’ earned income and FAS were significant (*p* < 0.001), ranging from 0.26 to 0.46 depending on examination year. For mothers’ earned income and FAS, the correlation coefficients (*r*) were also significant (*p* < 0.001) and varied between 0.26 and 0.41. For total parental earned income and FAS, correlation coefficients varied between 0.31 and 0.48 (*p* < 0.001).

**Table 4:** Associations (Pearson’s correlation coefficient, r) between parents’ earned income (2014–2018) and adolescents’ responses to the Family Affluence Scale (2015–2019)

Year	Father’s income	*n*	Mother’s income	*n*	Total parental income	*n*
2015	0.43	*93*	0.41	*98*	0.48	*89*
2016	0.46	*466*	0.28	*476*	0.44	*460*
2017	0.26	*640*	0.26	*659*	0.31	*633*
2018	0.28	*739*	0.34	*766*	0.35	*733*
2019	0.33	*203*	0.41	*214*	0.43	*201*

Note. All correlations were statistically significant (*p* < 0.001)

### Associations between parents’ level of education and FAS

Second, the association between parents’ level of education and adolescents’ scoring on the FAS was examined. As the results in Table 3 show, there was a relationship between parental level of education and median values of FAS. Among adolescents whose mother had attended only primary school, the median value of FAS was 8. Among those whose mothers had attended secondary school, the median value of FAS was 9. The highest median value of FAS, 10, was found among adolescents whose mothers had attended post-secondary school. The pattern was less pronounced when looking at fathers’ level of education and median values of FAS, where median values were 9 among those with only primary or secondary school education and 10 among those with post-secondary education. The results from the Kruskal-Wallis tests showed significant differences (*p <* 0.001) in FAS across both mothers’ and fathers’ educational levels (96.18 for mothers and 85.15 for fathers).

Spearman’s correlations were performed with parental level of education (1−3) and FAS as a continuous variable (0–13). The correlation coefficient (*r*) was 0.21 (*p* < 0.001) for fathers’ level of education and 0.19 (*p* < 0.001) for mothers’ level of education. Additional analyses using the original seven levels of education available in registers resulted in similar correlation coefficients (data not shown).

### Associations between parents’ occupational status and FAS

Third, the association between parental occupational status and adolescents’ scoring on the FAS was examined. As the results in Table 3 show, the lowest median value of FAS was found among adolescents without working parents (7), followed by adolescents with one parent working (9) and those with both parents working (10). The results from the Kruskal-Wallis test showed significant differences in FAS between the occupational statuses (110.14, *p* < 0.001).

Spearman’s correlations were also performed with parental occupational status (1−3) and FAS as a continuous variable (0–13), resulting in a correlation of 0.22 (*p* < 0.001).

### The functioning of current guidelines for low, medium and high FAS in Sweden

The second research question regarded the current guidelines from the HBSC network [25, 26], more precisely if the age-group and gender-specific ridit-based cut-offs for low, medium and high FAS are applicable among adolescents in Sweden.

The results (see Table 5) showed that the higher earned income mothers and fathers had, the lower the proportion of adolescents categorized as low FAS and the higher the proportion categorized as high FAS. For instance, among adolescents whose parents belonged to the *lowest* income quintile, 28.9% were categorized as low FAS, 65.2% as medium FAS and only 5.9% as high FAS. Among adolescents whose parents belonged to the *highest income* quintile, only 1.9% were categorized as low FAS, 65.8% as medium FAS and 32.3% as high FAS. Similar results were found for mothers’ and fathers’ earned income separately and adolescents categorized into low, medium and high FAS.

**Table 5:** Level of parental earned income, level of education and occupational status (2014–2018) and adolescents’ distribution after low, medium and high FAS (2015–2019)

	Low FAS (0–7) % (95% Confidence Intervals)	Medium FAS (8−11) % (95% Confidence Intervals)	High FAS (12−13) % (95% Confidence Intervals)	*n*
*Mother’s Earned Income*				
Quintile 1	**31.7** (27.5–36.1)	**61.1** (56.5–65.5)	**7.2** (5.1–10.0)	*445*
Quintile 2	**16.9** (13.7–20.7)	**74.6** (70.4–78.4)	**8.5** (6.2–11.4)	*449*
Quintile 3	**10.0** (7.5–13.2)	**76.6** (72.4–80.3)	**13.4** (10.5–16.9)	*440*
Quintile 4	**7.7** (5.6–10.6)	**75.3** (71.1–79.1)	**17.0** (13.8–20.7)	*442*
Quintile 5	**3.7** (2.3–5.9)	**70.3** (65.8–74.3)	**26.1** (22.2–30.4)	*437*
*Father’s Earned Income*				
Quintile 1	**29.6** (25.5–34.1)	**63.4** (58.7–67.8)	**7.0** (4.9–9.8)	*429*
Quintile 2	**17.9** (14.6–21.8)	**72.8** (68.4–76.8)	**9.3** (6.9–12.4)	*430*
Quintile 3	**8.4** (6.1–11.4)	**81.8** (77.8–85.1)	**9.8** (7.3–13.0)	*428*
Quintile 4	**6.7** (4.6–9.4)	**75.5** (71.2–79.4)	**17.8** (14.5–21.8)	*421*
Quintile 5	**2.5** (1.4–4.5)	**68.1** (63.6–72.3)	**29.3** (25.2–33.8)	*433*
*Total Parental Earned Income*				
Quintile 1	**28.9** (24.8–33.4)	**65.2** (60.5–69.6)	**5.9** (4.0–8.5)	*425*
Quintile 2	**17.2** (13.9–21.1)	**72.4** (68.0–76.4)	**10.4** (7.8–13.6)	*424*
Quintile 3	**8.8** (6.4–11.9)	**82.2** (78.3–85.6)	**9.0** (6.6–12.1)	*422*
Quintile 4	**6.4** (4.4–9.1)	**77.1** (72.9–80.9)	**16.5** (13.3–20.3)	*424*
Quintile 5	**1.9** (1.0–3.7)	**65.8** (61.1–70.2)	**32.3** (28.0–36.9)	*421*
*Mother’s Highest Level of Education*				
Primary School	**34.4** (27.3–42.3)	**60.3** (52.3–67.7)	**5.3** (2.7–10.1)	*151*
Secondary School	**16.9** (14.3–19.8)	**71.2** (67.8–74.4)	**12.0** (9.8–14.6)	*718*
Post-secondary school	**9.9** (8.4–11.6)	**73.3** (70.9–75.6)	**16.8** (14.9–18.9)	*1334*
*Father’s Highest Level of Education*				
Primary School	**28.0** (21.7–35.2)	**64.9** (57.4–71.7)	**7.1** (4.1–12.1)	*168*
Secondary School	**15.4** (13.1–18.1)	**74.2** (71.0–77.1)	**10.4** (8.5–12.7)	*805*
Post-secondary school	**8.5** (6.9–10.4)	**72.8** (69.9–75.6)	**18.7** (16.4–21.3)	*957*
*Parents’ occupational status*				
No parent works	**50.9** (38.3–63.4)	**47.4** (35.0–60.1)	**1.8** (0.3–9.3)	*57*
One parent works	**27.8** (22.7–33.5)	**63.3** (57.3–69.0)	**8.9** (6.0–13.0)	*259*
Both parents work	**9.3** (8.0–10.7)	**74.7** (72.6–76.6)	**16.1** (14.4–17.8)	*1800*

The results were similar for parental education level and FAS; the higher educational level mothers and fathers had, the lower the proportion of adolescents categorized as low FAS and the higher the proportion categorized as high FAS.

The results were clear for parental occupational status and FAS. Half (50.9%) of adolescents whose parents were not working were categorized into low FAS, and only 1.8% were categorized into high FAS. At the same time, only a fraction, 9.3%, of those with both parents working were categorized into low FAS. Note, however, that only 57 adolescents had two parents who were not working, while the large majority, 1800 adolescents, had two working parents.

**Fig. 2:**
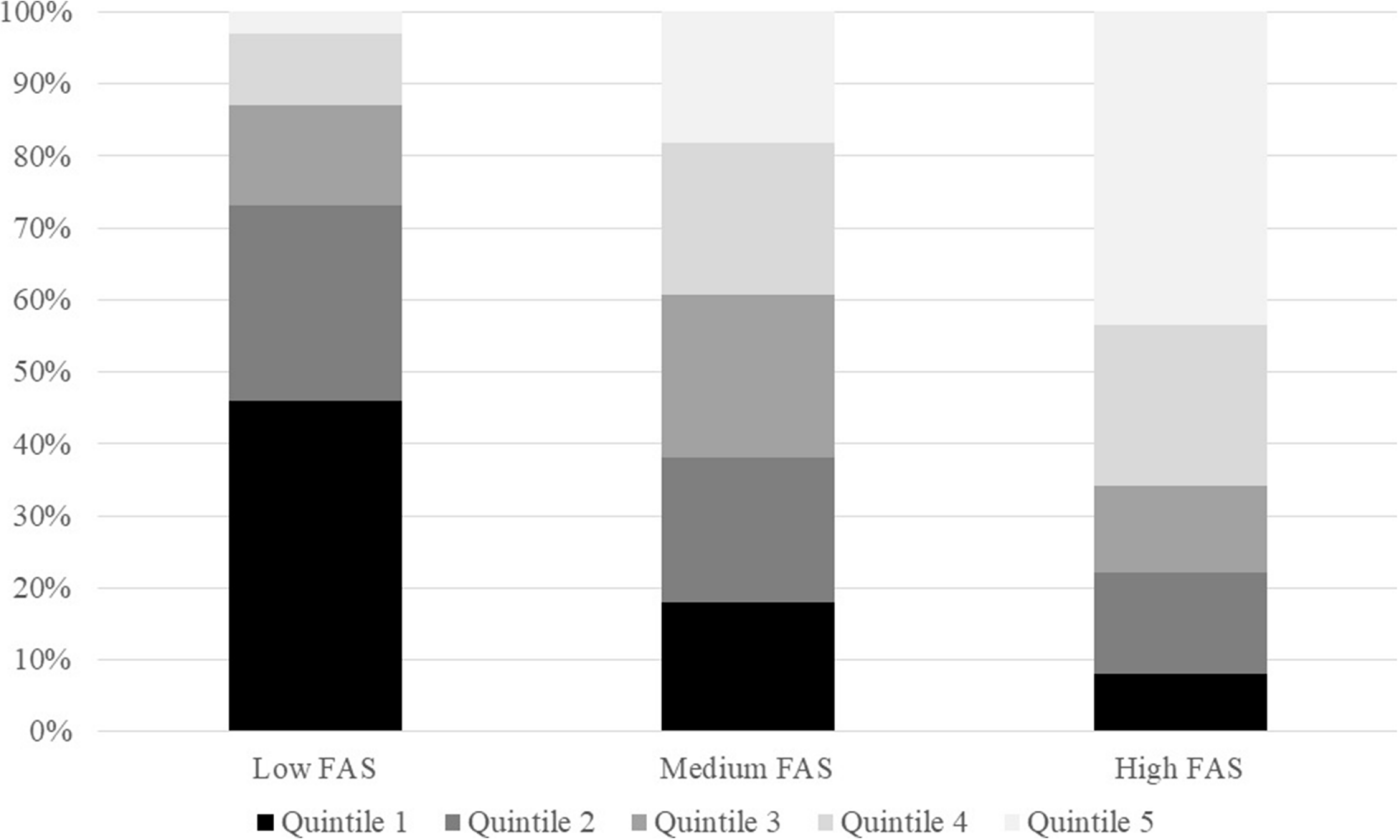
Levels of total parental earned income (2014–2018) in low, medium and high FAS groups (2015–2019)

Finally, the parents’ total earned income in the low, medium and high FAS groups was examined (see Fig. 2). As expected, the low FAS group consists mainly of adolescents with parents belonging to the two lower income quintiles (73.1%), while the high FAS group mainly consists of adolescents with parents belonging to the two higher income quintiles (65.8%). In contrast, in the medium FAS group, parents from each of the income quintiles are almost equally represented (18.0 to 22.6%).

## Discussion

This study has established that there are moderate associations between parents’ total earned income according to registers and adolescents’ responses to FAS. The associations found in this study are of similar magnitude as the associations found by Torsheim et al. 2016 [8], who examined the external validity of the FAS with parent-reported income in eight countries. They found that FAS scores were associated with parent-reported income in all of them, with an Eta-squared close to 0.30 (which could be interpreted as r^2^, which would give r = 0.55) in most countries. It is, however, not clear how parent income was categorized in the study by Torsheim et al. 2016 [8], why caution is necessary when making comparisons of the correlations.

The associations between parents’ level of education, occupational status and FAS were weak. A difference in parental level of education and occupational status was mainly observed between the low FAS group and the other two groups (medium and high FAS). The weak associations are in line with some of the previous studies examining the external validity of the scale using child-reported parental education, which have shown low [12, 14] or moderate external validity [15], but not with [13], who showed high external validity. Studies using child-reported occupational status have also shown low [14, 16] or moderate [3, 4] external validity.

The current HBSC guidelines for categorizing children and adolescents into low, medium and high FAS work in Sweden in such a way that the low FAS group mainly consists of adolescents whose parents belong to the two lowest income quintiles, while the high FAS group mainly consists of adolescents whose parents belong to the two highest income quintiles. In contrast, in the medium FAS group, parents from each of the income quintiles are almost equally represented. As it reflects *average* parental income, the medium FAS group may preferably be used as the reference group in analyses using low, medium and high FAS groups.

This study also showed that the internal reliability of the FAS was low (α = 0.516). This is a somewhat higher internal reliability than in the previous validation studies which have shown low internal reliability (0.310 ≤ α ≤ 0.401) [4, 12, 14], except for [15] who showed α = 0.580. However, they are based on the previous version of the FAS consisting of four items.

### Strengths and limitations

The main strength of the study is the high quality of STARS, where researchers have recruited the students and been present at schools during the data collection, along with the high quality register data regarding different dimensions of parental SES that have been linked to the adolescents in STARS as it contains personal identity numbers. Register data are more reliable than both child-reported and parent-reported data on income, education and occupation, which have been used in previous validation studies. Furthermore, missing data for both adolescents and parents were low. Additionally, all adolescents completed the FAS in the questionnaire. The risk of systematic bias in the results is therefore low.

The main limitation of this study is that the survey data come from a regional sample of adolescents, with a response rate of 45% and comprising only 13-year-olds. However, comparisons of the sample with the nationally representative HBSC study show only minor differences with regard to migration background and family structure. Register data for parental educational level showed that parents in STARS were slightly more educated than the population in general, which implies that adolescents with less educated parents may be somewhat underrepresented in STARS.

Another limitation is the use of *earned*, and not *disposable*, income in the analyses. In Sweden, all households with children receive monthly child benefits, and individuals who have low income may apply for housing allowances and income support to maintain a decent standard of living. Additionally, single-parent households normally receive allowances from the other parent. Last, earned income is the income before taxation. Taxation in Sweden is progressive, meaning that the higher the income, the higher the tax. As a result of transfers and progressive taxation, families’ disposable incomes are not equal to earned income, and this may especially apply to households at the lower end of the income distribution, which may receive transfers and pay lower taxes. There is a variable in registers showing the household’s disposable income/standard of living, where the household’s total disposable income is related to the household composition (number of adults and children) and current values for a decent standard of living. However, initial checks of this variable’s distribution showed many extreme values, as well as a low correlation to earned income. These findings made us concerned about the reliability and quality of the variable. A variable showing the absolute level of disposable income in SEK may have been more useful and reliable but was not available.

Please note that the STARS survey data were collected before the COVID-19 pandemic; thus, the data on the number of holidays abroad during the past 12 months were not affected by COVID-19.

## Conclusions

The Family Affluence Scale can mainly identify low- and high-income households in Sweden. It may be used as an alternative measure of total parental earned income in studies using self-reported socioeconomic status among adolescents.

Ensuring that instruments intended to measure socioeconomic inequalities in health are reliable is crucial in public health. The results in this study add to previous knowledge regarding the Family Affluence Scale, as it has not previously been validated using register data for parental socioeconomic status.

### Recommendations for future research

Future revisions and validations of the Family Affluence Scale are of course necessary, as living conditions improve, innovations are introduced and patterns of consumption change. Additionally, more countries use the Family Affluence Scale and need to ensure that it works in their country.

## Data Availability

The datasets generated and analysed during the current study are not publicly available because the STARS study comprises sensitive personal data and the register data from Statistics Sweden, which have been linked to the STARS study, contain personal data. The data are therefore protected by the Swedish Public Access and Secrecy Act and are not available upon request. However, researchers who are interested in collaboration on the STARS data should contact the principal investigator Professor Peter Friberg (peter.friberg@mednet.gu.se).
